# CHATTIER WITH FRIENDS: OLDER ADULTS’ DAILY SOCIAL CONTACT AND CONVERSATION

**DOI:** 10.1093/geroni/igz038.614

**Published:** 2019-11-08

**Authors:** Yee To Ng, Meng Huo, Karen Fingerman

**Affiliations:** 1 University of Texas, Austin, Austin, Texas, United States; 2 The University of Texas at Austin, Austin, Texas, United States

## Abstract

Studies suggest conversation improves cognitive skills among older adults. While contact with family members is common in late life, contact with friends and acquaintances is relatively less frequent. Yet, we know little about how often older adults engage in conversation when they have contact with different social partners. This study used data from the Daily Experiences and Well-being Study to investigate how older adults talk with different social partners on a daily basis. Participants (N = 303) completed an initial interview about their social partners and reported on their contact with each social partner in ecological momentary assessments every 3 hours across 5 to 6 days. Participants also wore Electronically Activated Recorders (EAR), which captured snippets of their daily conversation. Findings revealed that contact with family members (e.g., spouse, children, siblings) occurred most often, with less frequent contact with other social partners (e.g., acquaintances, neighbors), and then friends. Multilevel models also revealed that participants talked more (i.e., saying more words in each 30-second snippet and had a greater proportion of snippets when they talked) when they had contact with their friends than when they had contact with family members or other social partners. Results from these multiple methods suggest that daily contact with friends could potentially encourage conversation that may facilitate cognitive functioning among older adults.

